# Metabolic Silencing via Methionine-Based Amino Acid Restriction in Head and Neck Cancer

**DOI:** 10.3390/cimb45060289

**Published:** 2023-05-24

**Authors:** Anna Chiara Wünsch, Elena Ries, Sina Heinzelmann, Andrea Frabschka, Peter Christoph Wagner, Theresa Rauch, Corinna Koderer, Mohamed El-Mesery, Julian Manuel Volland, Alexander Christian Kübler, Stefan Hartmann, Axel Seher

**Affiliations:** 1 Department of Oral and Maxillofacial Plastic Surgery, University Hospital Wuerzburg, D-97070 Wuerzburg, Germany; wuenschanna@outlook.de (A.C.W.); elena.ries@icloud.com (E.R.); sina.heinzelmann@gmx.de (S.H.); andrea.frabschka@yahoo.de (A.F.); peterchristophwagner@t-online.de (P.C.W.); theresa.rauch98@web.de (T.R.); corinna.koderer@posteo.de (C.K.); julian_volland@gmx.de (J.M.V.); kuebler_a@ukw.de (A.C.K.); hartmann_s2@ukw.de (S.H.); 2 Department of Biochemistry, Faculty of Pharmacy, Mansoura University, Mansoura 35516, Egypt; m_elmesery@mans.edu.eg

**Keywords:** amino acid restriction, caloric restriction, methionine, HNSCC, SCCHN, cisplatin, amino acid transporter, SLC-family, cell vitality, low energy metabolism

## Abstract

In recent years, various forms of caloric restriction (CR) and amino acid or protein restriction (AAR or PR) have shown not only success in preventing age-associated diseases, such as type II diabetes and cardiovascular diseases, but also potential for cancer therapy. These strategies not only reprogram metabolism to low-energy metabolism (LEM), which is disadvantageous for neoplastic cells, but also significantly inhibit proliferation. Head and neck squamous cell carcinoma (HNSCC) is one of the most common tumour types, with over 600,000 new cases diagnosed annually worldwide. With a 5-year survival rate of approximately 55%, the poor prognosis has not improved despite extensive research and new adjuvant therapies. Therefore, for the first time, we analysed the potential of methionine restriction (MetR) in selected HNSCC cell lines. We investigated the influence of MetR on cell proliferation and vitality, the compensation for MetR by homocysteine, the gene regulation of different amino acid transporters, and the influence of cisplatin on cell proliferation in different HNSCC cell lines.

## 1. Introduction

Head and neck squamous cell carcinoma (HNSCC) is one of the most common tumour types, with over 600,000 new cases diagnosed annually worldwide. With a 5-year survival rate of approximately 55%, the poor prognosis is constant and has not truly improved, despite extensive research and the introduction of new adjuvant therapies [1,2]. For this reason, the development of new strategies in cancer therapy is of enormous importance.

One of the essential characteristics of neoplastic cells is their unlimited proliferation, which results in a space-occupying lesion. For successful proliferation, two components must be available in sufficient-energy and mass. Energy is available as ATP/NADH, and mass is available as amino acids; the main components of cell mass [3] are enzymes, structural proteins, and precursors and intermediates of numerous metabolic molecules. In recent years, various forms of restriction have become increasingly important; among these strategies, the approach, which we refer to as “metabolic silencing”, leads to the induction of so-called low energy metabolism (LEM). If one continuously limits energy in the form of caloric restriction (CR) [4] or mass by amino acid or protein restriction (AAR or PR) [5], the cell switches to an economical mode, which leads to the inhibition of proliferation and the induction of autophagy at the cellular level [6,7]. Such restrictions have extremely positive effects when applied permanently. The most important are the extension of lifespan and the reduction and prevention of age-associated diseases such as type II diabetes, cardiovascular diseases, and cancer. Surprisingly, this principle applies across species from yeast to Drosophila, rodents, and humans [4,5].

Moreover, the mechanisms can be explained very well. Complex networks such as hormones and growth factors, as well as the interactions between individual organs from the brain to the liver, are of great importance [8]. However, many of the decisive processes within the individual cell take place at the molecular level. A multitude of sensors continuously measure energy and mass. The ratio of AMP to ATP is measured by AMP kinase (AMPK) [9], the NAD+ level is measured via sirtuins [10], and the contents of selected amino acids (e.g., leucine, arginine, glutamine, serine, and methionine) are measured via various protein complexes. SAMTOR, for example, indirectly measures methionine content via the intermediate product S-adenosylmethionine (SAM) [11,12]. Vitamins and their intracellular availability can also have a significant influence on the proteome and its activity [13]. All these signals then transmit to the essential intracellular switching centre—the mechanistic target of rapamycin (mTOR). The sum of the signals mTOR receives determines whether mTOR actively promotes proliferation/growth or whether the cell switches to LEM by inhibiting proliferation and activating autophagy [11,14,15].

In principle, it is possible to exert a targeted influence on this network and induce LEM by means of metabolic silencing. Thus, a permanent reduction in calories by 10% to 50% or a reduction in the amount of protein is not always necessary; the restriction of a selected amino acid is sufficient to set in motion a process that is almost identical to the mechanisms at work in CR or PR. Due to the central position of the amino acid methionine, methionine restriction (MetR) is often used [16,17]. Although MetR is easy to implement in everyday laboratory work, e.g., by simply removing methionine from the cell culture medium, it is much more difficult to implement as a therapy since methionine must be either enzymatically degraded in vivo (by means of the bacterial enzyme methionase) or removed from ingested food [18,19]. Basically, MetR in vitro is only representative of energy or mass restriction, also known as metabolic silencing, which is a universal and methodical approach within tumour therapy. MetR shows the enormous potential of the AAR and allows molecular biological processes to be analysed in the laboratory, but it is in no way mandatory that this approach be implemented in vivo.

In principle, AAR and PR have great potential in cancer therapy. Levine et al. [20] impressively demonstrated the influence of PR on the progression of tumours in mice via in vivo experiments. Thus, in mice fed a lower amount of protein (4–7%) compared to the control group, the size of the developing tumours was drastically reduced (by approx. 80%), and in some of the mice (approx. 20%) the development of tumours was completely suppressed, although 10,000 aggressive neoplastic cells were inoculated [20].

In this work, for the first time, we analysed the potential of MetR to inhibit proliferation in selected HNSCC cell lines. In addition, the cell lines were tested for their methionine dependence. Although methionine itself is an essential amino acid, methionine can be regenerated at the cellular level from various intermediates, such as homocysteine (Hcy). Tumour cells very often lose this ability, which may enhance the effect of MetR as a tumour therapy [21]. We also tested the influence of MetR on cell vitality, the gene regulation of different amino acid transporters, and the influence of cisplatin on cell proliferation in different HNSCC cell lines.

## 2. Materials and Methods

### 2.1. Cell Culture

The murine fibroblast cell line L929 was purchased from the Leibniz Institute, DSMZ-German Collection of Microorganisms and Cell Cultures GmbH (Braunschweig, Germany). The human cell lines HeLa and HaCaT, and the HNSCC cell lines FaDu, Detroit562, SCC9, and SCC25 were purchased from the American Type Culture Collection (ATCC)(LGC Standards, Wesel, Germany). L929 and HeLa cells were cultured in RPMI 1640 medium (Gibco, Life Technologies; Darmstadt, Germany), HaCaT cells were cultured in DMEM (Gibco, Life Technologies), FaDu and Detroit562 cells were cultured in MEM alpha (Gibco, Life Technologies), and SCC9 and SCC25 cells were cultured in DMEM/F12 (Gibco, Life Technologies). All cell lines were cultured with 10% FCS (Sigma–Aldrich, Darmstadt, Germany) and 1% penicillin/streptomycin (P/S; 100 U/mL penicillin and 100 µg/mL streptomycin (Thermo Fisher Scientific, Darmstadt, Germany)). Media for SCC9 and SCC25 cell lines were additionally supplemented with 25 mM HEPES (Thermo Fisher) and 10 µL/L hydrocortisone (400 ng/mL) (Sigma–Aldrich). All cells were cultured at 37 °C in a humidified atmosphere containing 5% CO_2_. For the AAR experiments, media lacking the amino acid methionine was used. For the controls (full medium), the amino acid methionine (Sigma–Aldrich, Darmstadt, Germany) was added at the concentrations indicated as follows: RPMI 1640 medium (Genaxxon, Ulm, Germany) 15 mg/L L-methionine; MEM alpha (Bio&Cell, Feucht, Germany) 15 mg/L L-methionine; DMEM (Gibco, Life Technologies) 30 mg/L L-methionine; and DMEM/F12 (Genaxxon) 17.25 mg/L L-methionine.

### 2.2. Crystal Violet Staining

Cells were seeded at 10,000 cells in 100 µL of culture medium per well of a 96-well plate and incubated overnight. The following day, the cells were incubated in a full or methionine-free (Met(-)) medium. For compensation experiments, DL-Hcy (Sigma–Aldrich) was used at 800 µM and 1.5 µM vitamin B12 (Sigma–Aldrich) in Met(-) medium. The number of measured values, the incubation period, and the number of experimental repetitions are mentioned in the corresponding figure legends. For staining, the supernatants were removed, and the cells in each well were incubated with 50 µL of crystal violet solution (1% crystal violet in 20% methanol; Carl Roth, Karlsruhe, Germany) for 10 min and subsequently washed five times with distilled water. The plates were dried for 2 h in the dark. For quantification, 100 µL of methanol was added to each well, and the plate was incubated for 10 min until the crystal violet was completely dissolved. The photometric absorbance was measured at 595 nm using a microplate reader (Tecan, Crailsheim, Germany). For data analysis, the experiments were repeated at the indicated times to calculate the mean values and standard deviations. The results were normalised to the untreated control (100%). The relative cell number values determined via the crystal violet assay with the stimulated probes (CV_S_) were normalised to those of the untreated control (CV_C_) ((CV_S_/CV_C_) = CV_R_). To obtain the percentage values, the CV_R_ value was multiplied by 100 (RCN (%) = (CV_S_/CV_C_) × 100 = CV_R_%).

### 2.3. ImageXpress Pico Automated Cell Imaging System—Digital Microscopy (Pico Assay)

Cells were seeded at 10,000 cells in 100 µL of culture medium per well of a 96-well plate and incubated overnight. The following day, the cells were incubated in a complete or methionine-free medium. The incubation time is stated in the corresponding figure legends. For staining, 10 µL of Hoechst staining solution (1:200 dilution of Hoechst 33342 (Thermo Fisher, Darmstadt, Germany) (10 mg/mL in H_2_O) in medium) was added to each well. After a 20–30 min incubation period, wells were analysed with an ImageXpress Pico automated cell imaging system (Molecular Devices, San Jose, CA, USA) via automated digital microscopy. The cells were analysed with transmitted light and in the DAPI channel at 4× magnification. The complete area of every well was screened. The focus and exposure time were set via auto setup and controlled by analysing 3–4 test wells. Finally, every result was confirmed visually, and 95% of cells were counted and analysed.

### 2.4. Analysis of the Cell Progression Rate Using the Pico Assay

Cells were seeded at 10,000 cells in 100 µL of culture medium per well in a 96-well plate. After 24, 31, 48, 55, 72, 79, 96, and 103 h, cell numbers were measured with six values for every time point as described under the Pico Assay Section (2.3). The growth of a cell population can be described with the following formula:

$$N_{t}=N_{0}\times 2^{\left(t\times f\right)}$$

(*N_t_* = cell number at time *t*; *N*_0_ = cell number at time 0; *t* = time in days (d); *f* = cell division frequency (1/d)).

To determine *f*, the formula is rearranged as follows:

$$f=\frac{\left(log\left(\frac{N_{t}}{N_{0}}\right)/log(2)\right)}{t}$$

To obtain an overview, the measured values were first plotted as a simple diagram. From this, it was possible to see at what point the growth entered the plateau phase. From these values, the individual *f* values were calculated for the intermediate periods (e.g., Δ24/32, Δ32/48). The total value *f* was then calculated as the mean of the Δ*f* values.

### 2.5. Live/Dead Assay

The cell lines L929, HeLa, HaCaT, Detroit562, FaDu, SCC9, and SCC25 were seeded at 10,000 cells/well on a 96-well plate. On the following day, the medium was removed, and the cells were stimulated with a full medium or Met(-) medium. Cells stimulated with 1 µM staurosporine (Seleckchem, Planegg, Germany) served as the death control in each case. After 6 h, 24 h, and 48 h, measurements were performed with the EarlyTox Live/Dead Assay Kit (Molecular Devices). For this, the medium was removed, and 100 µL/well of a staining solution (5 µg/mL Hoechst 33342 (company), 6 µM EthD-III, and 6 µM CAM in PBS (company)) was added per well. After 30 min of incubation, measurements were made using the “Cell Scoring: 3 Channels” program of the ImageXpress Pico Automated Cell Imaging System (Molecular Devices). To determine the absolute cell number, measurements were made in transmitted light and in the DAPI channel using Hoechst 33342, as well as via the FITC (CAM) and TexasRed (TRITC)/(EthD-III) channels over the entire area of the respective well at 4x magnification. Exposure time and focus were determined by auto settings. Subsequently, for better visualisation of the cells, the measurement was repeated with a selected area of the well (1.39 mm × 1.39 mm) at 10× magnification, from which the images shown in this publication were selected. For each experimental condition, 2 wells were evaluated. The experiment was performed two times.

### 2.6. Semiquantitative RT–PCR

A total of 500,000 cells/well were seeded in a 6-well plate. The following day, cells were stimulated with or without methionine in the corresponding medium for 24 h and 72 h. RNA isolation was performed using an RNeasy Mini Kit (Qiagen, Hilden, Germany) according to the instructions provided by the manufacturer. One microgram of RNA was transcribed into cDNA using the QuantiTect Reverse Transcription Kit (Qiagen) according to the instructions provided by the manufacturer. Next, 20 ng cDNA was used in the PCR with 1.5 µL of the appropriate primer (QuantiTect Primer Assay, Qiagen) and 12.5 µL of a ready-to-use qPCR master mix (QuantiTect SYBR Green PCR Kit, Qiagen). The thermal cycling program was composed of initial denaturation at 95 °C for 15 min, 39 cycles at 95 °C for 15 s, 30 s at 54 °C, and 30 s at 72 °C. Triplicates for each data point were measured. The β-actin and GAPDH genes were used as internal controls (standard). The following primers from Qiagen were used: ACTB QT00095431, GAPDH QT00079247, SLC7A5 (LAT1) QT00089145, SLC7A11 QT00002674, SLC1A5 QT00083909, and SLC43a2 QT00027559. For analysis, the 2^−ΔΔ^Ct method for relative quantification of gene expression was used, including error calculation (range) based on Livak and Schmittgen [22].

### 2.7. Statistical Analysis

Statistical analysis was performed using the GraphPad Prism program (version 6.04; GraphPad Software, San Diego, CA, USA). One-way ANOVA was used to compare and analyse data of different groups, followed by the Tukey–Kramer multiple comparison test (ns; nonsignificant; * *p* < 0.05, ** *p* < 0.01; *** *p* < 0.001).

## 3. Results

As already mentioned, the proliferation of neoplastic cells is an essential characteristic. For this reason, we analysed the influence of MetR on the proliferation of the established HNSCC cell lines FaDu, Detroit562, SCC9, and SCC25 in initial experiments. For comparison, the two human cell lines HeLa, generated from cervical carcinoma cells, and HaCaT, an immortalised keratinocyte line, were used. To assess the potential of AAR, the murine fibroblast cell line L929, which was established as a model system for MetR in earlier work by our research group, was also used [23].

### 3.1. Restriction of Methionine Inhibited the Proliferation of HNSCC Cell Lines

**Figure 1:**
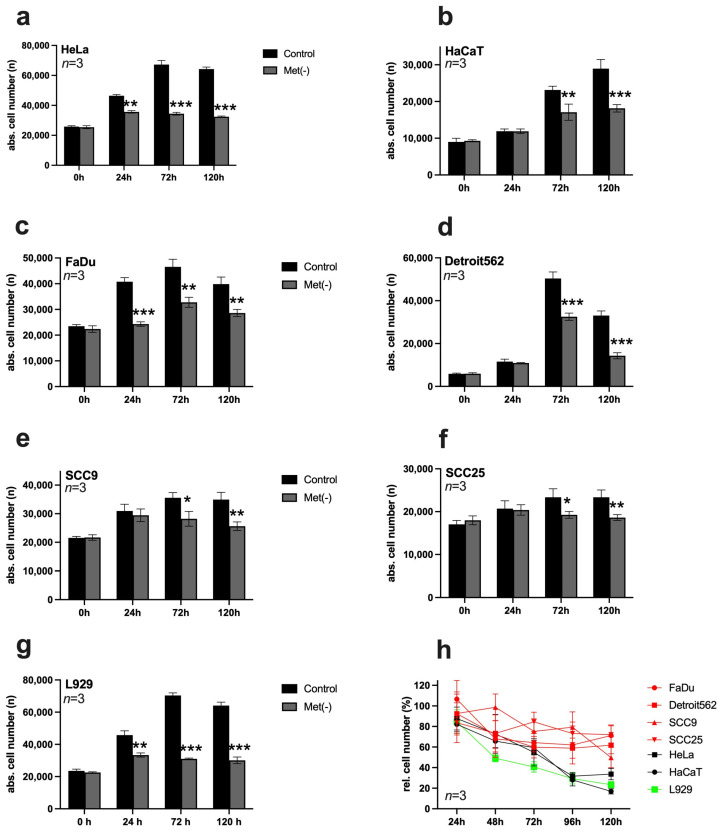
Analysis of proliferation in different cell lines subjected to restriction of the amino acid methionine. (**a**–**g**) In each case, 10,000 cells/well were seeded on 96-well plates and stimulated in triplicate on the following day with a control medium or Met(-) medium. Absolute cell counts were analysed at 0 h, 24 h, 72 h, and 120 h using digital microscopy with the ImageXpress Pico automated cell imaging system. A summary of the results from three experimental replicates (*n* = 3) is shown in each case. (**h**) Summary of the proliferation analysis based on relative cell number counted using crystal violet staining. In each case, 10,000 cells/well were seeded on 96-well plates and stimulated the following day with a control medium (set as 100%) or Met(-) medium. Cells were analysed at 24 h, 48 h, 72 h, 96 h, and 120 h. A summary of the results from three experimental replicates (*n* = 3) is shown in each case (* *p* < 0.05, ** *p* < 0.01; *** *p* < 0.001).

In each case, 10,000 cells/well were seeded on 96-well plates and stimulated the following day with a control medium or Met(-). At time points of 0 h, 24 h, 72 h, and 120 h, the cells were analysed by digital microscopy using the ImageXpress Pico automated cell imaging system (Figure 1a–g). Then, the DNA of the cell nuclei was stained with Hoechst 33342, allowing automated counting of the stained nuclei over the entire area of the well and, thus, determination of the absolute cell number. In HeLa cells (Figure 1a), MetR significantly inhibited proliferation at 24 h, while in HaCaT cells (Figure 1b), the effect was recognisable after 72 h. In essence, MetR had an antiproliferative effect in HNSCC cells that was observed at 24 h in FaDu cells (Figure 1c), and significant after 72 h in Detroit562 (Figure 1d), SCC9 (Figure 1e), and SCC25 cells (Figure 1f). Although MetR had a clear effect in all HNSCC cell lines analysed, a comparison of the results with those obtained in the murine cell line L929 (Figure 1g) clearly showed that the effect took more time and was less pronounced in these cell lines than that in the control. This became clear in the overview of the analysed cells (Figure 1h). Crystal violet staining, a simple and fast method, was used here for the analysis. In this case, data from the control group at the respective time were set as 100%, and data from the Met(-) group are shown relative to these baseline data. Although only relative cell numbers could be determined in this way, this experiment allowed a better representation of the dynamics of MetR. It became clear that the murine cell line (green line) reacted most effectively to MetR. The two human cell lines (black lines) showed almost equal efficiency, but the response in these cells was delayed compared to that of L929 cells. All HNSCC cells (red lines) responded to MetR, but after an initial decrease in proliferation, the cells entered a plateau phase again, indicating that they could at least partially or temporarily compensate for the effects of MetR.

### 3.2. Analysis of the Cell Progression Rate

**Figure 2:**
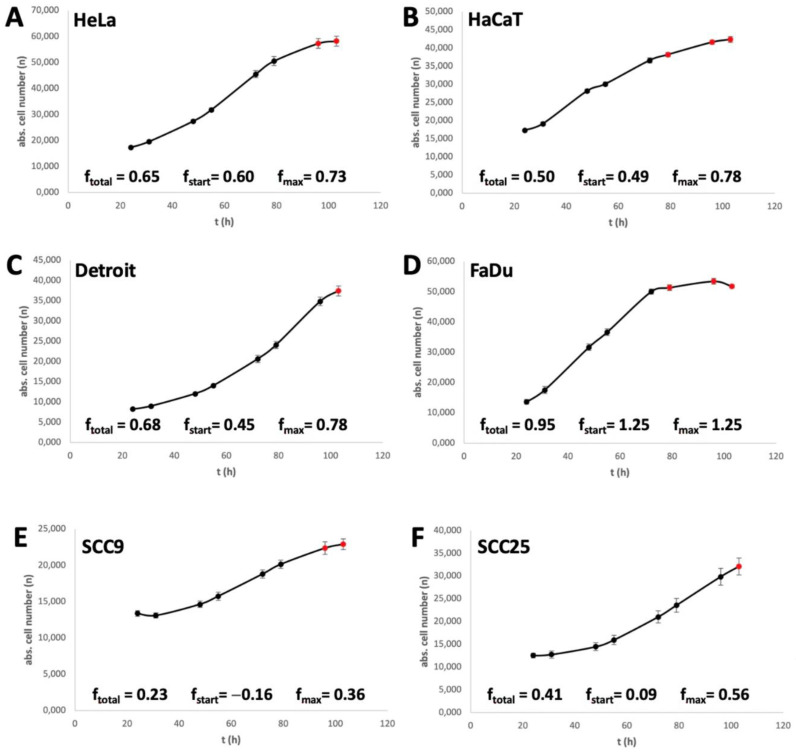
Analysis of the cell progression rate. (**A**–**F**) Cells were seeded at 10,000 cells/well in a 96-well plate. After 24, 31, 48, 55, 72, 79, 96, and 103 h, cell numbers were measured as described in Section 2 under Pico Assay (2.3). The summarised results (*n* = 3) are shown in Figure 2.

The different speeds at which cells react to a MetR are influenced by many factors, from the possibility of storing methionine to regenerating the molecule via detours, e.g., autophagy. Another factor is the proliferation rate of the cell. As a simple example, if a cell line does not divide within the first 24 h after splitting in culture, no effect of MetR on the proliferation rate can be determined. In addition, the speed at which a cell divides plays a role; this speed is referred to here as the f value. The f value indicates the frequency with which a cell or a cell population divides/doubles with a simple numerical value. A value of 1 means that the population doubles once within one day. Thus, we determined the f value for the cell lines used, as previously published [24]. Three different f values were determined for further analysis. The value f_total_ indicates the division rate over the entire period investigated until cell division enters the saturation phase. In Figure 2, the measured values used for this analysis are marked in black, and the excluded measured values are marked in red. The value f_start_ indicates the division rate at the beginning of the experiment within the first 7 h, and f_max_ is the highest f value achieved within the measurement period.

It is easy to see that the f values and the response rate to MetR correlate within a certain range. This is particularly clear for the cell lines SCC9 and SCC25, which have the lowest f values (Figure 2E,F) and the lowest response rate to MetR (Figure 1e,f). On the other hand, FaDu, with the highest f values, also has the corresponding best MetR response rate. In HaCaT and Detroit cells, the low MetR sensitivity seems to be due to the slow f_start_ values (0.49 and 0.45) at the beginning of proliferation. Here, the cells have to go until the amino acid restriction is reflected in the division behaviour. On the other hand, the HeLa cell line starts proliferating relatively quickly and responds to MetR at an early stage (Figure 1a and Figure 2A).

### 3.3. HNSCC Cell Lines Are Mainly Methionine Dependent

**Figure 3:**
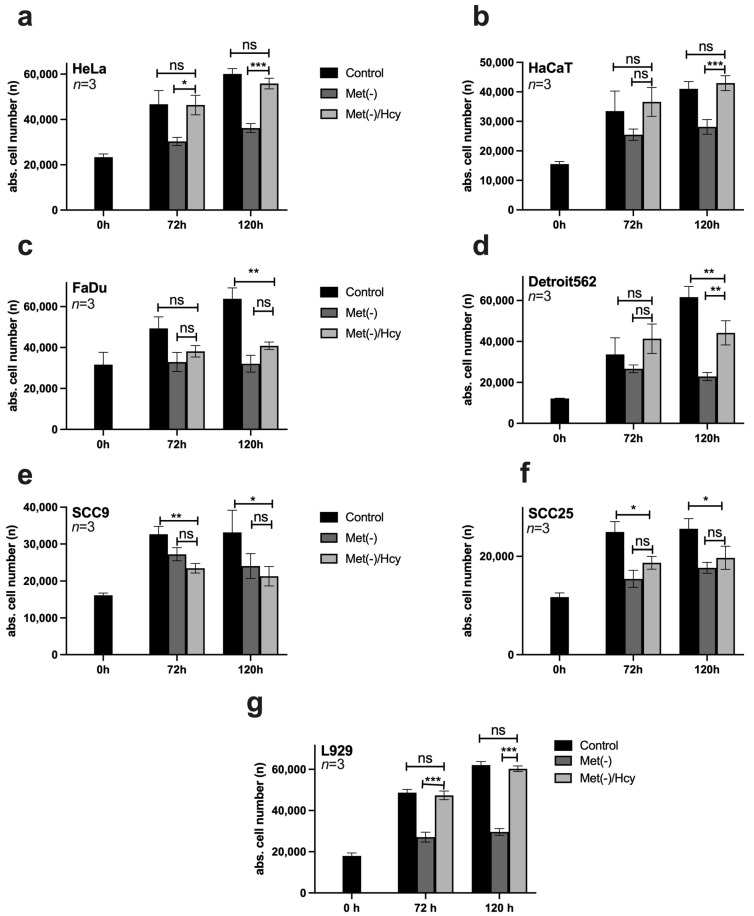
Homocysteine (Hcy)-based competition of MetR in different cell lines. (**a**–**g**) In each case, 10,000 cells/well were seeded on 96-well plates and stimulated in triplicate on the following day with control medium, Met(-) medium, or Met(-) medium to which 800 μM Hcy had been added (Met(-)/Hcy). Cells were analysed at 0 h, 72 h, and 120 h using the ImageXpress Pico automated cell imaging system to determine absolute cell counts. A summary of the results from three experimental replicates (*n* = 3) is shown in each case (ns; nonsignificant; * *p* < 0.05, ** *p* < 0.01; *** *p* < 0.001).

Methionine is one of the most important amino acids, partly because of its roles as an amino acid required for the initiation of protein expression and a central molecule in metabolism. Thus, methionine can generally be regenerated by cells from various intermediates, such as SAM or Hcy, to a limited extent [25]. However, tumour cells often lose this ability [21]. For this reason, the methionine dependence of the selected cell lines was analysed in a compensation experiment. In each case, 10,000 cells/well were seeded on 96-well plates and stimulated on the following day with control medium, Met(-) medium, or Met(-) medium to which 800 μM Hcy had been added (Met(-)/Hcy). Cells were analysed at 0 h, 72 h, and 120 h using the ImageXpress Pico automated cell imaging system to determine absolute cell numbers. The results for HeLa and HaCaT cells show a compensatory response to MetR via Hcy to 100% (Figure 3a,b). HNSCC cells, on the other hand, had difficulty compensating for MetR with Hcy, and no significant compensation was measured in FaDU, SCC9, and SCC25. Furthermore, only Detroit562 shows homocysteine-mediated compensation after 120 h, which does not correspond to the growth of the control. (Figure 3c–f). For completeness, the results of the compensation experiment in L929 cells are also shown (Figure 3g).

### 3.4. MetR Did Not Induce Significant Cell Death

**Figure 4:**
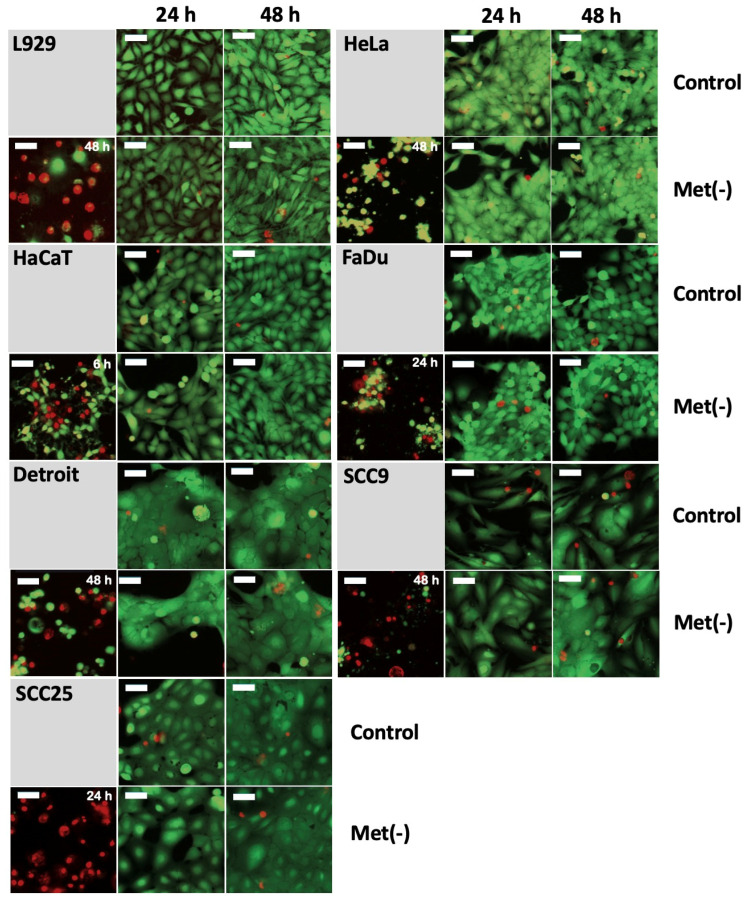
The cell lines L929, HeLa, HaCaT, Detroit562, FaDu, SCC9, and SCC25 were seeded at 10,000 cells/well on a 96-well plate. On the following day, the medium was removed, and the cells were stimulated with full or Met(-) medium. Cells stimulated with 1 µM staurosporine served as the dead control cells. After 6 h, 24 h, and 48 h, measurements were performed with the “EarlyTox Live/Dead Assay Kit” (Molecular Devices). Measurements were made using the “Cell Scoring: 3 Channels” program of the ImageXpress Pico Automated Cell Imaging System (Molecular Devices) with a 10× magnification. The cytoplasm of leaving cells was measured in the FITC channel with CAM, and dead cells were measured in Texas Red-(TRITC)-channel with EthD-III. The results for 24 h and 48 h are shown. The 6 h time point showed no cell death for the control or MetR group. The dead cell control is on the left side for every cell line, and the time of the dead cell control measurement is indicated in the upper right corner. The size of the white bar corresponds to 52.44 µm. For each experimental condition, two wells were evaluated. The experiment was performed two times independently. The representative results of one experiment are shown. For better resolution, the figures are also added as a supplement (Suppl. Figure S1—Live/Dead Assay Figures).

In principle, the proliferation studies under MetR (Figure 1) do not directly prove that the reduced cell number is due to a purely antiproliferative mechanism and not to a form of cell death. For L929 cells, we have already shown in a previous publication that MetR is purely antiproliferative and does not induce significant cell death [23]. In this work, we analysed cell viability using the live/dead assay. As shown in Figure 1h, a significantly reduced cell number can already be detected after 48 h for most of the cell lines used here. For this reason, cell viability was analysed at the early time points of 6, 24, and 48 h, as the beginning stages of cell death should already be detectable during this period. It is necessary to analyse the early time points because at the later time points, dead cells can dissolve (apoptotic bodies or autophagy), or after death, adherent cells can sphere off, detach from the cell culture matrix, and go into suspension; thus, these cells are no longer clearly detectable via live/dead staining. The two dyes, calcein AM (CAM) and ethidium homodimer-III (EthD-III), were used for analysis by digital cell microscopy. CAM is a membrane-permeable dye. In living cells, the nonfluorescent CAM is converted into green, fluorescent calcein by means of acetoxymethyl ester hydrolysis by intracellular esterases. Thus, the majority of the cytoplasm is stained green, which, in addition to the transmitted light analysis, allows an additional optical analysis of the cell shape, since dying or dead cells usually change shape considerably and eventually sphere off. EthD-III is a red fluorescent dead cell stain for bacteria and mammalian cells. It is a cell membrane-impermeant nucleic acid dye that stains only dead cells with damaged cell membranes. Staurosporine (1 µM), a nonspecific inhibitor of various protein kinases (e.g., PKA and PKC) that can induce cell death, was used as a positive control [26]. Figure 4 shows the results for the time points 24 and 48 h for all cell lines examined. While staurosporine was able to induce increased cell death in all cell lines, the comparison between control and MetR shows neither an increase in cell death under amino acid restriction nor a significant change in cell shape, which would indicate that the cell is approaching cell death. The cells showed the same vitality as in the control.

### 3.5. Under MetR, Amino Acid Transporters Are Subsequently Upregulated Then Downregulated

Basically, amino acids are one of the most essential resources for cell growth and proliferation, and as already mentioned, they are responsible for the largest part of the cell mass [3]. Membrane-bound amino acid transporters (AAT), which allow the import and export of metabolites, are correspondingly important. The families of AATs are large and quite heterogeneous. The substrate specificity is just as varied as the transport mechanisms, which exploit all possibilities from UniProt to symport to antiport [27]. In this work, we focused on the gene regulation of four AATs that are very frequently analysed in the context of cancer. These are SLC7A5 (LAT1), SLC7A11 (Xc- or xCT system), SLC1A5 (ASCT2), and SLC43a2 (LAT4). SLC7A5 mediates the uptake of neutral AAs (leucine, isoleucine, phenylalanine, methionine, histidine, tryptophan, valine, and tyrosine) into cells in exchange for the efflux of intracellular substrates (AAs and/or glutamine). System Xc(-) is composed of a light-chain subunit xCT (also known as SLC7A11) and a heavy-chain subunit (CD98hc, also referred to as SLC3A2) and functions as a Na^+^-independent transporter that mediates the exchange of extracellular cystine for intracellular glutamate. SLC1A5 serves as an obligatory exchanger that imports a sodium-coupled amino acid substrate into cells and exports another sodium-coupled amino acid substrate with 1:1 stoichiometry. It is the primary transporter for importing glutamine. Glutamine flux, which is dependent on the balance between the uptake of glutamine by SLC1A5 and its subsequent export by SLC7A5, leads to high intracellular availability of essential amino acids (EAAs) (overview of SLC7A5, SLC7A11, and SLC1A5 in [28]). The amino acid transport activity induced by SLC43a2 (LAT4) is sodium-, chloride- and pH-independent. The main amino acids transported are isoleucine, leucine, phenylalanine, and methionine [29]. This transporter is of particular importance because it is upregulated in neoplastic cells and thus enables methionine to be “stolen” from the environment to increase the growth of the tumour [30,31].

**Figure 5:**
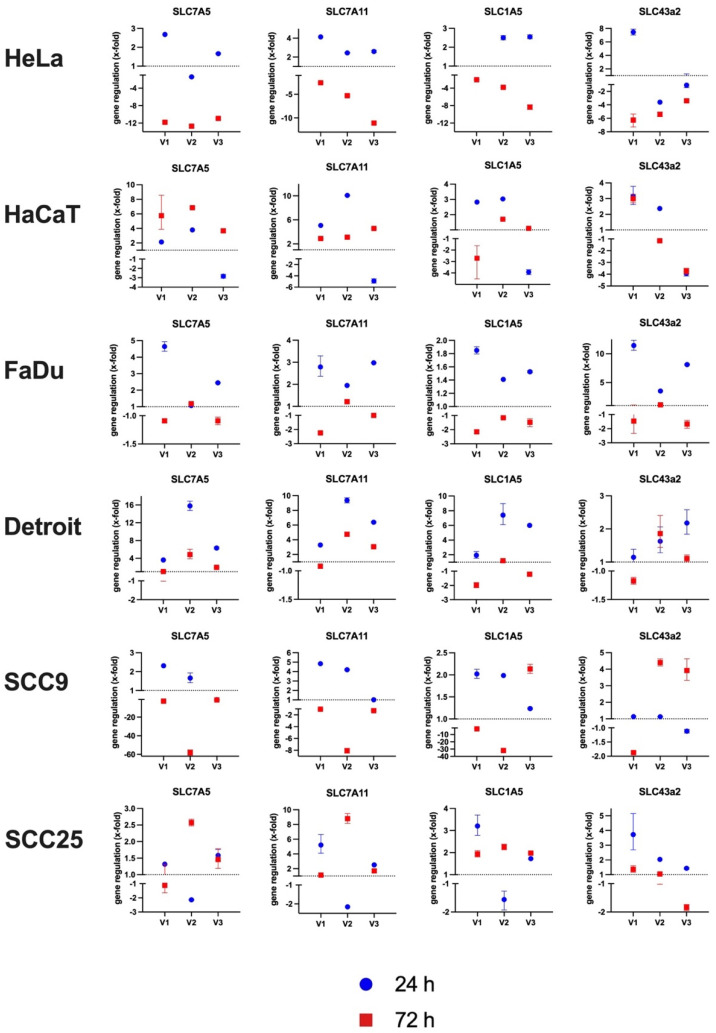
Analysis of the gene expression of different amino acid transporters. The cells were stimulated for 24 h or 72 h with or without methionine in the corresponding medium. The isolated RNA was transcribed into cDNA, and the relative expression was determined by RT–PCR using the 2^−ΔΔ^Ct method. β-Actin was used as a standard. The results of the relative expression of the samples under MetR are shown. The dashed line represents an unchanged expression at a value of 1. The total of three independent experiments is shown individually, with the corresponding value of each experiment for 24 h and 72 h. Each PCR value was determined as a triplet. The range (error range) of each measured value is marked by error bars. An overview of the numeric results is attached as a table in the supplement (Suppl. S2—RT_PCR_Results_Total).

The gene expression of the mentioned AAT was examined in the cell panel under MetR after 24 h and 72 h by RT–PCR. The experiment was repeated three times (V1–V3). Figure 5 shows the differential expression (x-fold) in comparison to the control at the same time point in the individual experiments. An overview of the numeric results is attached as a table in the supplement (Suppl. S2—RT_PCR_Results_Total).

The results show no clearly significant, strictly reproducible results for the genes studied. However, in most cases, it can be observed that within the first 24 h, the receptors are upregulated, and the cells respond to the amino acid restriction with an increased expression compared to the control, while after 72 h, the expression then decreases again. This can be seen very well in the cell line HeLa for the receptor SLC7A11. For the cell line SSC9, on the other hand, the results for all analysed genes are very heterogeneous and do not show a clear trend.

### 3.6. The Efficacy of Cisplatin Is Only Marginally Affected by MetR

**Figure 6:**
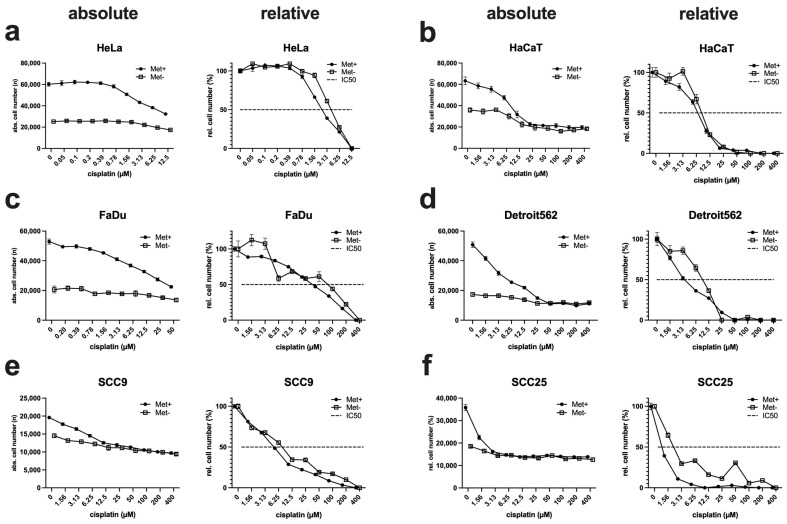
Analysis of the efficacy of cisplatin under MetR. (**a**–**f**) A total of 10,000 cells/well were seeded on 96-well plates. On the following day, cells were incubated in triplicate with control or Met(-) medium and a log2 dilution of cisplatin, with a starting concentration of 400 µM (12.5 µM for HeLa and 50 µM for FaDu). After 72 h, the cells were analysed using the ImageXpress Pico automated cell imaging system to determine absolute cell numbers. To compare the efficacy of cisplatin under different conditions, we used the IC_50_, which is defined as the concentration at which the drug reaches its half-maximal efficacy. For this purpose, the absolute cell counts (shown on the left side for each cell line) must be converted into percentages (shown on the right side for each cell line). The respective control value (without cisplatin) was set as 100%, and the lowest value in a measurement series was set as 0%. If one now places the two curves in a diagram, one can see whether there is a shift in the curve and, thus, a change in the IC_50_. For objective representation, the results of the absolute cell numbers are shown for each cell line, as well as the normalised representation in percent to the right. The dashed line indicates the inhibition of proliferation at 50% (IC_50_). The experiments were carried out three times. The figures show a summary of the experiments (*n* = 3) in one diagram.

In the field of HNSCC therapy, the classic cytostatic drug cisplatin plays a very important role, as the range of adjuvant drug therapies has hardly improved in recent decades [32]. For this reason, we analysed the influence of MetR on the efficacy of cisplatin. Thus, cells were incubated for 72 h with a cisplatin log2 dilution series in full medium and under MetR, and subsequently evaluated using the ImageXpress Pico automated cell imaging system to determine absolute cell numbers. To compare the efficacy of cisplatin under different conditions, we used the IC_50_, which is defined as the concentration at which the drug reaches its half-maximal efficacy. For this purpose, the absolute cell numbers must be converted into percentages. Therefore, the control value (without cisplatin) was set as 100%, and the lowest value in a measurement series was set as 0%. If one now places the two curves in a diagram, one can see whether there is a shift in the curve and, thus, a change in the IC_50_. For objective representation, the results of the absolute cell numbers are shown for each cell line, as well as the normalised representation in percent to the right (Figure 6). If one compares the individual IC_50_ values with each other, one can see that the values are either almost identical or increase only slightly under MetR. As a trend, the curves are almost identical at the beginning and at the end, which means that the basic sensitivity of the cells to cisplatin changes only marginally. The slight “deterioration” of the IC50 can be explained simply by the mechanism of action of cisplatin. Cisplatin is incorporated into newly synthesised DNA during the initial phase of cell division and causes DNA cross-linking, which then leads to cell death via different mechanisms [33]. Cells under MetR, in which proliferation is inhibited and DNA synthesis comes to a standstill, accordingly show an increase in the IC_50_ of cisplatin. This may seem counterintuitive in the context of chemotherapy, but we show in the discussion that this is not the case precisely because of the phenomenon of “differential stress resistance.”

## 4. Discussion

In principle, different forms of restriction have great potential to prevent the development of age-associated diseases such as type II diabetes, cardiovascular diseases, and cancer if they are applied continuously. At the same time, they exert their effects through many common mechanisms at the molecular level, which, as already mentioned, are, above all, reduced proliferation, the induction of autophagy and the implementation of LEM. For this reason, restriction is, in principle, also an interesting approach in tumour therapy. The work of Levine et al. [20,34] in suppressing the development and progression of tumours in vivo in mice through protein restriction, as well as the successful use of different caloric restriction mimetics (CRMs), shows enormous potential.

In this paper, for the first time, we publish the effects of AAR on the proliferation of HNSCC cell lines. Regarding proliferation, the restriction of methionine had a significant effect on proliferation and the proliferation rate. Strikingly, the proliferation of the four HNSCC cell lines FaDu, Detroit562, SCC9, and SCC25 reached a plateau, and restriction did not show the same efficiency in the other cell lines examined (Figure 1h). In essence, cancer cells are characterised by metabolic programming; in other words, they optimise their cellular metabolism to the conditions to enable growth and proliferation. One of the best-known mechanisms is the Warburg effect [35]. It is, therefore, not surprising that tumour cells can resist restriction to a certain extent. More importantly, all HNSCC cell lines showed a response to AAR within the first 72 h.

The results regarding Hcy compensation are also promising. All four HNSCC cell lines showed a lower ability or no ability to compensate for methionine with Hcy, which is in line with the consensus in the literature [21]. This may also be why the absolute number of HNSCC cells decreased slightly after a period of AAR. Of course, the complete absence of the amino acid methionine inevitably leads to cell death after a certain period of time. In contrast, cell death by MetR did not play a significant role and was not induced in the first 48 h in any cell line used in this publication, as shown by the live/dead assay (Figure 2). The decreasing cell numbers in some cell lines after 120 h can usually be attributed to the consumption of essential metabolites (e.g., glucose) in the medium, as the total cell number decreased both in the control and under MetR.

The most likely reason, however, for the lower influence of MetR on the proliferation rate in HNSCC cell lines is simply the time factor. A short impulse is probably not sufficient to have a lasting and significant influence on proliferation, which occurred with a delay and was not as efficient as in HaCaT or L929 cells, for example. We have already observed this phenomenon in many other experiments, which is why medium- and long-term experiments on AAR in HNSCC seem to be necessary. A very good approach here is the use of perfusion cultures, which make it possible to supply cells with fresh medium over a longer period permanently, and thus also investigate long-term effects on cells in culture. We used this method in another work to analyse the influence of MetR compared to glucose restriction over a longer time period on the murine cell line L929 [24]. A further argument for the necessity of long-term analyses in the form of perfusion culture is the differential expression results for the amino acid transporters. In the short term, the different amino acid transporters were upregulated after 24 h, but then generally downregulated after 72 h (Figure 5). Only an analysis over several days under constant conditions in the perfusion culture can show whether this is permanent.

However, the experiments clearly show that no extreme stress situation occurs due to MetR, but the cell can react or act specifically to deficits of energy and mass with its programmes developed during evolution. The experiments with cisplatin also prove that MetR in vitro has no direct negative influence on the effectiveness of the drug.

The fact that there is no more extreme stress situation can also be seen in the expression analyses of the amino acid transporters, which tend to be upregulated in the short term but then down-regulated again. However, transcriptome analyses, which analyse a large number of genes simultaneously, would be helpful to show the exact reaction of a cell to an amino acid restriction at the transcription level.

In the immediate context of cancer, the question arises to what extent, for example, MetR can influence the effectiveness of various drugs and what influence the restriction has on the growth of the tumour. Basically, there are two possible situations. First, MetR forces the cells in the entire organism into a LEM. This would result in an inhibition of proliferation, which would also affect the tumour. In this case, the growth of the tumour would at least be limited. The second situation appears counterproductive at first glance. LEM is only induced in healthy tissues, and proliferation is also only limited to healthy cells. The neoplastic cells ignore the signals and are further programmed to divide at a high rate. It is exactly this situation that creates an extreme, which is of enormous advantage for tumour therapy. This situation is called “differential stress resistance” (DSR). Cancer cells that express oncogenes and exhibit egocentric proliferative behaviour respond to certain cancer-promoting and growth-promoting factors. Moreover, cancer cells do not respond to protective signals generated by short-term fasting (SCR) or long-term nutrient restriction. Hence, cells can be exposed to the following two different extreme situations: somatic cells may be protected, while cancer cells become increasingly vulnerable to attack [36].

A good example of this theory is the use of cisplatin in combination with fasting. Short-term starvation (STS) based on caloric and/or protein reduction protects normal cells while simultaneously sensitising malignant cells to high-dose chemotherapeutic drugs such as cisplatin in mice and possibly patients. The fasting-dependent protection of normal cells and sensitisation of malignant cells depend, in part, on reduced levels of IGF-1 and glucose [37].

We were able to show in this work for the first time that MetR represents a further promising alternative in HNSCC therapy. Furthermore, this approach is supported by the increased methionine dependence of HNSCC cell lines, which should enhance the efficiency of methionine-based therapy.

## 5. Conclusions

In this work, it was shown for the first time that methionine-based amino acid restriction represents a further promising alternative in HNSCC therapy. Furthermore, the use of this approach is supported by the increased methionine dependence of HNSCC cell lines, which should enhance the efficiency of methionine-based therapy.

## Data Availability

For better resolution, the figures of the live/dead assays are added as a PowerPoint file (Suppl. Figure S1—Live/Dead Assay Figures). An overview of the numeric results of the PCR experiments is attached as a prism table in the supplement (Suppl. S2—RT_PCR_Results_Total).

## Supplementary Materials

The following supporting information can be downloaded at: https://www.mdpi.com/article/10.3390/cimb45060289/s1 Figure S1. Live/Dead Assay Figures; Suppl. S2 RT_PCR_Results_Total.
