# Supplementary material for: Metabolic Silencing via Methionine-Based Amino Acid Restriction in Head and Neck Cancer
Source: Curr Issues Mol Biol. 2023 May 24;45(6):4557–73. doi: 10.3390/cimb45060289 (PMC10297726; doi:10.3390/cimb45060289)
Supplement: Supplementary file 1 [file cimb-45-00289-s001.zip › Suppl. 1 ΓÇô Live:Dead Assay Figures:s1.pptx]

## Slide 1
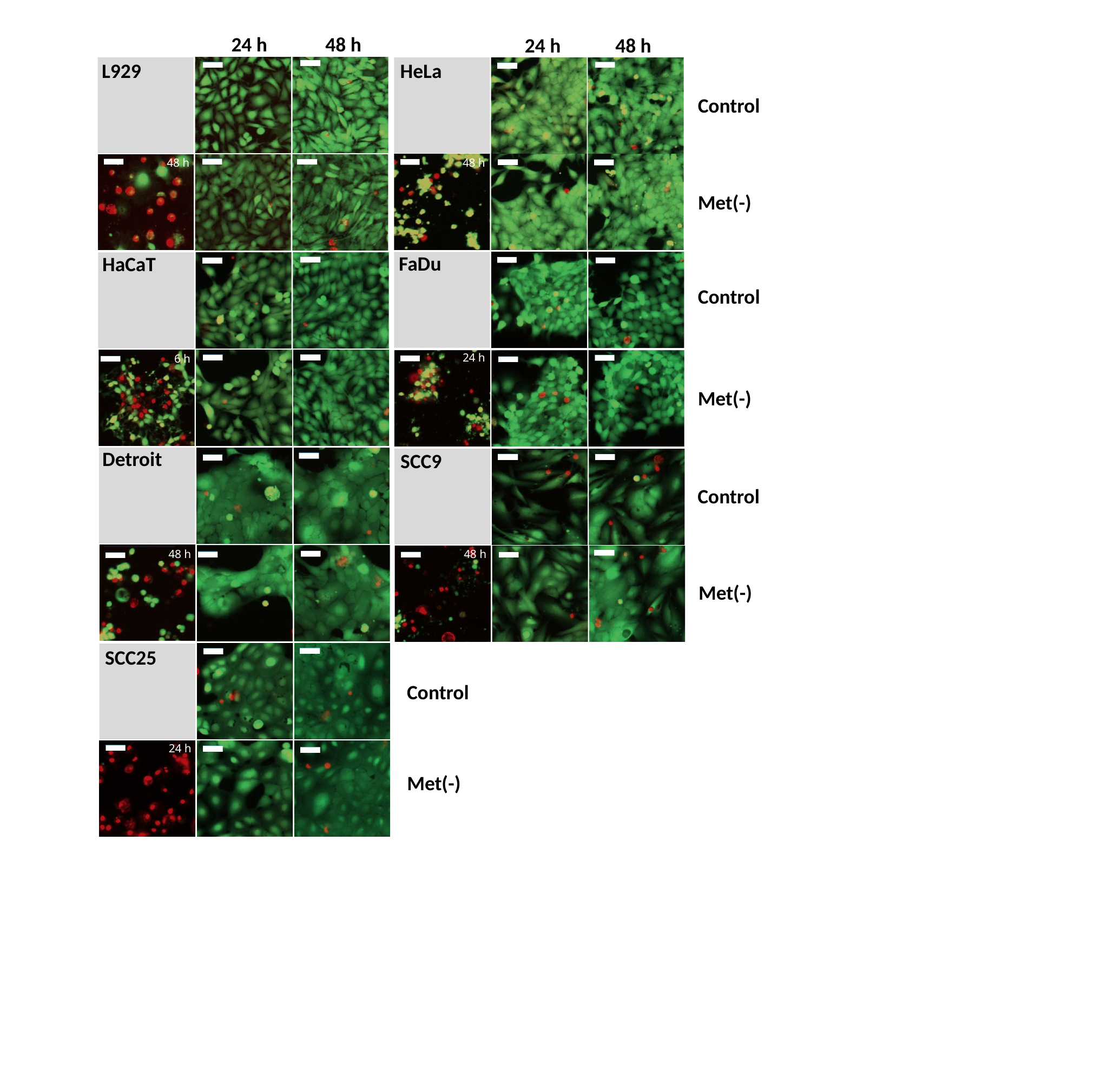

24 h
48 h
24 h
48 h
HeLa
L929
Control
48 h
48 h
Met(-)
FaDu
HaCaT
Control
24 h
6 h
Met(-)
Detroit
SCC9
Control
48 h
48 h
Met(-)
SCC25
Control
24 h
Met(-)
